# Incidental Gallbladder Neoplasms: A Growing Global Burden

**DOI:** 10.7759/cureus.25805

**Published:** 2022-06-09

**Authors:** Neha Singh, Ariba Zaidi, Rupinder Kaur, Jasleen Kaur, Vijay S Nijhawan

**Affiliations:** 1 Pathology, Maharishi Markandeshwar Institute of Medical Science and Research, Ambala, IND

**Keywords:** gallstone, radiology, incidental gallbladder carcinoma, histopathology, cholecystectomies

## Abstract

Background

The increasing trend of laparoscopic procedures has made cholecystectomies one of the most common surgical specimens received for histopathological evaluation. This has also led to an increasing trend of finding incidental gallbladder malignancies for a presumed benign disease. The present study describes the histopathological spectrum of neoplastic lesions of the gallbladder along with the historadiological correlation with special emphasis on incidental gallbladder carcinomas (IGBC).

Materials and methods

All the cholecystectomies received over a span of two and a half years were studied. Demographic details, imaging findings, gross features, and microscopic findings of premalignant and malignant lesions were noted. Special stains were done as and when required.

Results

Of the 1253 cholecystectomies received during the study period, 50 gallbladders (3.9%) showed neoplastic pathology and were included in the present study. The age range was 40 to 60 years with female predominance. Ultrasonography revealed nonspecific wall thickness in both premalignant and incidental gallbladder carcinomas. Gallstones were seen in 74% of the cases (37/50). Gross and imaging findings in 17 (34%) of the malignant cases were in concordance with microscopic features, whereas the dysplastic lesions (21) and IGBC cases showed evidence of chronic cholecystitis on the same. Microscopic examination revealed focal dysplasia (low and high grade) in 21/50 (42%) cases. Invasive malignancy was seen in 28/50 (56%) of the cases, of which 11 cases (22%) were IGBC. Pancreaticobiliary type of adenocarcinoma was the most common morphology seen in almost all the cases. There was also one case each of intracholecystic papillary neoplasm (ICPN) and carcinosarcoma.

Conclusion

GBC is an unusual malignancy and its preoperative diagnosis is not so definitive. The incidental form of GBC presents as a radiological disguise and a histopathological surprise. Hence, the present study warrants a complete and scrupulous histopathological examination of all the cholecystectomy specimens for proper and further management of the case.

## Introduction

Gallbladder (GB) malignancies, though rare, are one of the most common cancers of the biliary tract after bile duct carcinoma, having a worldwide incidence ranging from 0.3%-1.5% [1-3]. The incidence in India ranges from 0.8%-1% with central India having a higher incidence as compared to the southern part [4-5]. Around 70%-80% of GB malignancies show the presence of gallstones, and the risk of GB cancer in patients with the latter has been reported to have increased four to seven times [6-8]. GB malignancies and dysplastic lesions pose a big diagnostic dilemma both clinically and radiologically. They have overlapping features with benign conditions and about 0.2%-3.3% of them are diagnosed incidentally, either intraoperatively or on histopathological examination [4,9-11]. 

The present study describes the histopathological spectrum of neoplastic lesions of the gallbladder with a special emphasis on IGBC.

## Materials and methods

**Figure 1:**
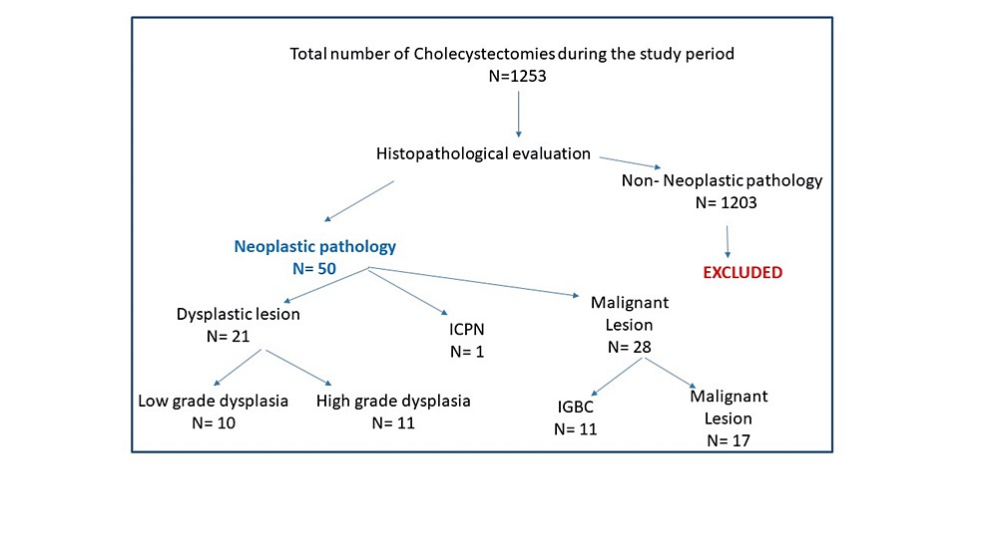
Flow chart showing the selection pattern of the cases ICPN = Intracholecystic papillary neoplasm, IGBC = Incidental gallbladder carcinoma, N = Number

This was a retrospective observational study done on neoplastic gallbladder lesions in the histopathology section of the department of pathology in a tertiary care center in North India. Details of a total of 1253 cholecystectomy specimens received during the study period of two and a half years (Jan 2019 to Aug 2021) were retrieved from departmental records. Patients with histopathological diagnoses of inflammatory as well as other non-neoplastic pathology were excluded from the study. Neoplastic pathology was reported in only 50 cases that were included in the study (Figure 1).

The detailed demographic profile, clinical details, and gross findings of all the 50 cases were retrieved both from histopathology request forms as well as from medical records. Clinical details were not available in most cases due to incomplete data recording in the record files.

Hematoxylin and Eosin (H&E)-stained slides were retrieved from the departmental records and were reviewed by the authors. Deeper sections, special stains, and immunohistochemistry (IHC) were performed as and when required.

The tumor, nodes, and metastases (TNM) classification was done for all the malignant cases.

Statistical analysis

The data were entered in an Excel sheet (Microsoft Corporation, Redmond, WA) and analyzed using SPSS software version 20 (IBM Corp., Armonk, NY). Categorical variables were expressed as frequencies (%) and mean.

## Results

A total of 1253 cholecystectomies were subjected to histopathological examination during the study period. Only 50 cases with neoplastic pathology were included in the study. The age group of all the lesions ranged between 40 and 60 years (average: 55 years). Females formed the majority (77.5%; 31/50) with a male to female ratio of 1:1.8. Clinically, all the patients presented with nonspecific symptoms like right upper quadrant pain abdomen and vomiting with the duration of illness ranging anywhere from two months to eight months.

Radiologically, all premalignant, as well as incidentally diagnosed malignant lesions (IGBC), showed features suggestive of chronic cholecystitis with cholelithiasis. However, 60.7% (17/28) of gallbladders revealed focal intraluminal or wall involvement by mass-like lesions with ill-defined margins and low echogenicity, thereby correlating with histological findings.

Gallstones were seen in 37/50 (74%) of the premalignant lesions, IGBC, and frankly malignant lesions on gross examination. There were non-specific changes like focal wall thickening seen in 31/50 (62%) of cases, ranging from 0.2 to 1.5 cm. Gross examination of clinically suspected malignant cases (17/50) revealed an ulceroinfiltrative to a proliferative growth pattern in the form of intraluminal growth.

Demographic, clinico-imaging, and gross features of all the cases have been provided in Table 1.

**Table 1:** Demographic, clinico-imaging, and gross features of all the cases CC = Chronic cholecystitis, CL = Chronic cholelithiasis, ICPN = Intracholecystic papillary neoplasm, IGBC = Incidental gallbladder carcinoma, NS = Nonsignificant.

		Dysplasia, n (%)		ICPN, n (%)	IGBC, n (%)	Malignancy, n (%)
No. of cases, n=50		Low grade	High grade	1 (2%)	11 (22%)	17 (34%)
		10 (20%)	11 (22%)			
Mean age (years)		42	45	41	47	48
M:F		1:2	1:2	1:0	1:1.5	0.6:1
Clinical features		pain abdomen and vomiting	pain abdomen and vomiting	pain abdomen and vomiting	pain abdomen and vomiting	pain abdomen and vomiting
Imaging features		CC &CL	CC &CL	CC &CL	CC &CL	Focal intraluminal and/ or wall involvement; mass-like lesion with ill-defined margins with low echogenicity
Gross features	Wall thickness (cm)	0.4- 0.6	0.4 to 0.6	0.5	0.4-0.8	0.8 cm -1 cm
	Morphology	NS	NS	Ulcero- proliferative lesion	NS	Ulceroinfiltrative to proliferative intraluminal growth.

**Figure 2:**
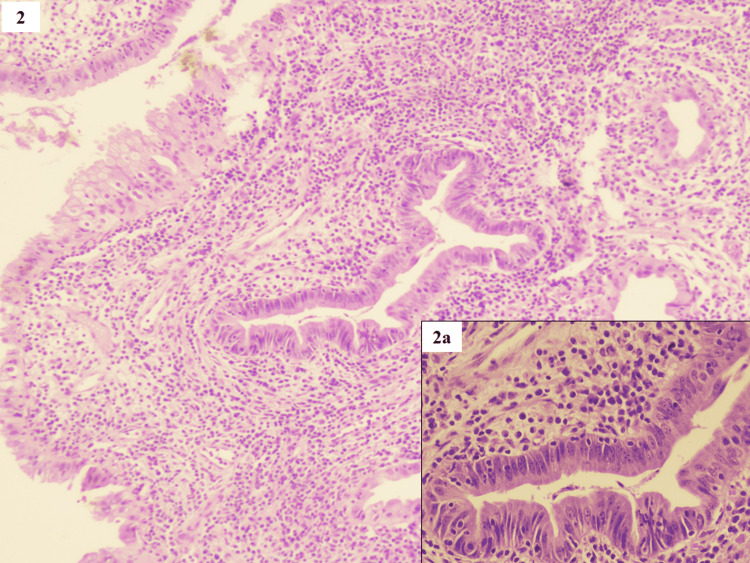
Low-grade dysplasia - confinement of the columnar cells toward the basal aspect of the epithelium with a mild nuclear enlargement (H&E, x100) Inset 2a shows nuclear pseudostratification, hyperchromasia, chromatin clumping, and irregular nuclear membrane (H&E, x 400)

Microscopically focal dysplastic changes were seen in 21/50 (42%) cases. Out of these, 10 cases revealed low-grade dysplasia and 11 cases revealed high-grade dysplasia focally. All the cases showed an abrupt transition from the normal epithelium to a dysplastic epithelium. Confinement of the columnar cells toward the basal aspect of the epithelium, with mild nuclear enlargement, hyperchromasia, chromatin clumping, irregular nuclear membrane, and pseudostratification, was seen in low-grade dysplastic lesions (Figure 2).

**Figure 3:**
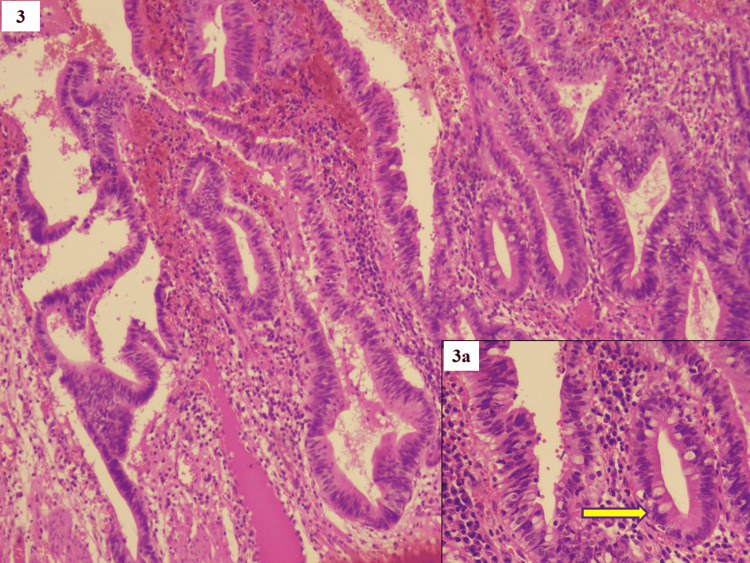
High-grade dysplasia - revealed marked nuclear enlargement, hyperchromasia, membrane irregularity, and apoptosis with significant loss of polarity (H&E, x100) Inset 3a shows intestinal metaplasia (yellow arrow) in the form of scattered goblet cells with basal pseudostratification of nuclei (H&E, x400).

High-grade lesions revealed marked nuclear enlargement, hyperchromasia, membrane irregularity, and apoptosis with significant loss of polarity. There was the presence of pyloric and intestinal metaplasia in the form of scattered goblet cells with basal pseudostratification of nuclei in 10/21 of the cases (Figure 3).

**Figure 4:**
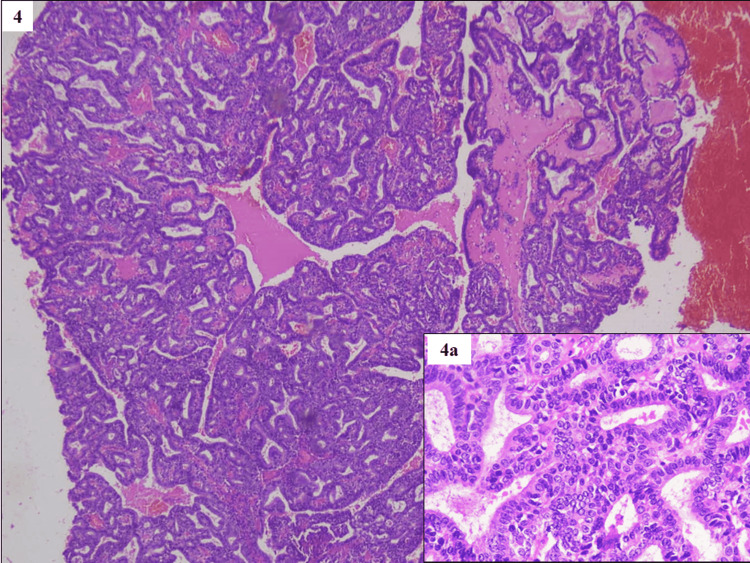
Intracholecystic papillary neoplasm (ICPN) and intraluminal growth of back-to-back epithelial units in a papillary or tubulopapillary configuration (H&E, x100) Inset 4a shows a high-power view (H&E, x400).

None of the cases showed monotonous appearing cells or cells with oncocytic features. There was no evidence of injuries like erosion, congestion, or stromal fibrosis corresponding to the area of dysplasia. Exhaustive sampling with a microscopic assessment of all the sections did not reveal any area of invasion. One interesting case of ICPN was also seen with microscopic features of intraluminal growth of back-to-back epithelial units in a papillary or tubulopapillary configuration (Figure 4).

**Figure 5:**
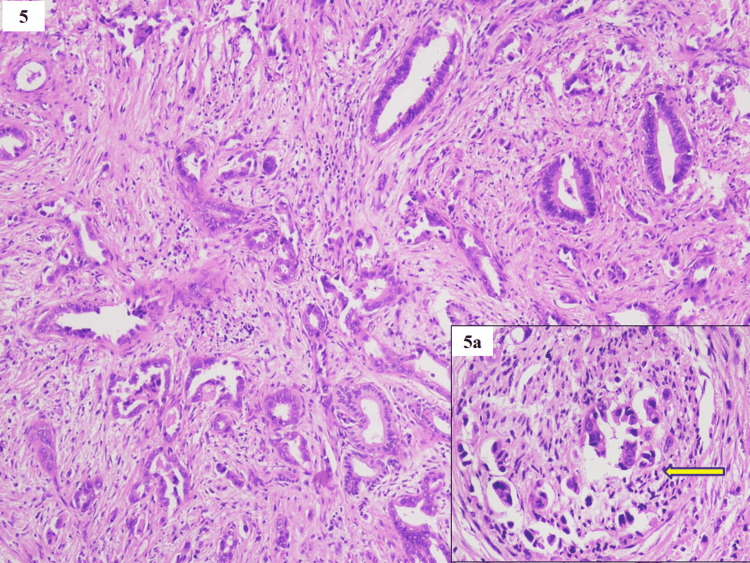
Adenocarcinoma - tumor cells formed irregular glands and papillae of variable size and shape with angulated contours in the desmoplastic stroma (H&E, x100) Inset 5a shows perineural invasion (yellow arrow) (H&E, x400)

Among the malignant cases, pancreaticobiliary adenocarcinoma was the most common invasive malignancy (27/28 cases) seen on histopathological examination (HPE). The tumor cells formed irregular glands and papillae of variable size and shape with angulated contours in the desmoplastic stroma. The cells were round to cuboidal with pleomorphic vesicular nuclei, prominent nucleoli, and moderate eosinophilic cytoplasm (Figure 5).

**Figure 6:**
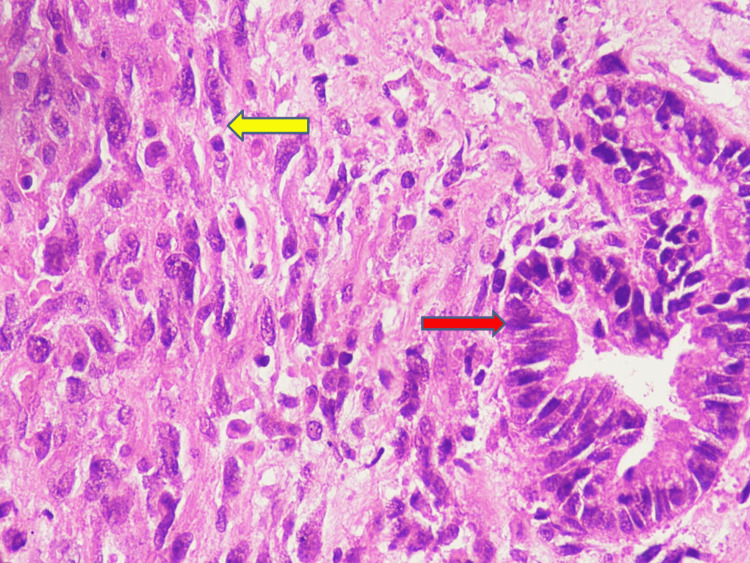
Carcinosarcoma - exhibiting carcinomatous (red arrow) and sarcomatous areas (yellow arrow) (H&E, x400)

One rare case of carcinosarcoma was also seen exhibiting adenocarcinomatous and fibrosarcomatous areas (Figure 6).

Out of the 17 frank malignant cases, 10 showed the involvement of serosa (pT3) and four cases revealed the involvement of the adjacent liver (pT3). Comment upon nodal status was not possible due to the non-availability of the same.

In cases of IGBC, the involvement of the lamina propria was seen in the majority, with four cases showing focal invasion into the muscularis propria (pT1). None of these cases showed the involvement of the serosa.

All the cases of IGBC were followed up till April 2022, of which mortality was documented in only one patient who also had other associated comorbid conditions.

## Discussion

The gallbladder is one of the most commonly resected specimens subjected to a histopathological examination. Cholelithiasis is the most common indication for surgery irrespective of whether the lesion is neoplastic or non-neoplastic [12]. Chronic cholecystitis is the most common benign lesion encountered on routine histopathological examination. GBC is an uncommon entity in the western world, however, it comprises the fifth most common cancer of the gastrointestinal and biliary tract malignancies and carries a dismal prognosis [4,7]. GBC across the globe has a wide geographical variation with a higher prevalence seen in South America, Eastern Europe, Japan, and some regions of India (more so along the Gangetic plains of northern India) while it is relatively rare in Northern Europe and America [5,10,13-15]. The prevalence of gallbladder malignancy in our study was seen in about 2.23% (28/1253) of all the cholecystectomies subjected to histopathological evaluation during the study period.

GBC is usually seen in the sixth to seventh decades of life and is two to six times more common among females as compared to males, which was comparable to the present study [2,4,16]. Gallstones are the major co-morbid risk factors seen in 70-98% of GBC cases with an incidence of GBC related to cholelithiasis ranging from 0.3% to 12%. [2,4,7,11,17-19]. Estrogen causing increased cholesterol super-saturation in the bile has been attributed to the gallstone-mediated GBC pathogenesis [20]. Gallstones were seen in 74% of all the neoplastic cases in the present study, which was in accordance with the above-mentioned studies.

The occurrence of precursor lesions in the gallbladder mucosa is often seen due to the injury-inflammation-regeneration-neoplastic transformation sequence and is most commonly attributed to irritation by gallstones [11]. Terminology like low and high-grade dysplasia has been used by some pathologists, however, the low-grade dysplasia is sometimes considered as an extension of marked reparative changes. High-grade dysplasia per the World Health Organization (WHO) has been labeled as biliary intraepithelial neoplasia grade 3’ (BilIN-3) so as to avoid overtreatment of such cases [21]. These lesions are usually associated with invasive malignancy in about 80% of the cases [22-23]. The present study showed 21 cases of dysplasia of which 11 exhibited focal high-grade dysplasia. However, none of the cases in our study showed invasive foci despite extensive tissue sampling.

IGBC is the terminology used when the GBC is diagnosed only on microscopic examination. The studies have shown its incidence ranging from 0.2%-3.3%. [1,4,9-11,24]. Out of the total who received cholecystectomies during the study period, 11 GBC (11/1253; 0.87%) were diagnosed incidentally in our study, which was in accordance with the published literature. Most of the IGBC in our study showed the presence of gallstones and varied wall thickness both on imaging as well as on gross morphology.

Imaging studies for GBC are usually seen either as a mass replacing the gallbladder (40%-65%) or focal or diffuse wall thickening (20%-30%) or it presents as an intraluminal polypoid mass in 15%-25% of the cases [25]. The present study showed imaging correlation in about 34% of the cases, which was comparable to the studies.

The pancreaticobiliary type of adenocarcinoma is the most common subtype encountered on HPE representing almost 90-95% of all the gallbladder malignancies followed by squamous or adenosquamous carcinomas [1-2,4,7- 8,10,12,14-15]. The majority of the malignant cases in the present study were also adenocarcinomas, which was in accordance with the above-mentioned studies.

Two rarer lesions, carcinosarcoma (one case) and ICPN (one case), were also seen in our study. Few authors have also shown the presence of these rarer lesions in their studies as a case report or mentioned in an article [12,26].

Limitations

Data presented in this study have been taken from a single institution with a limited number of cases, hence it cannot reflect the correct magnitude of the disease. Another drawback of this study was the non-availability of the relevant clinical laboratory parameters due to a lack of data in the record file. Complete follow-up data could be retrieved for IGBC cases only.

Disclaimer

The present study did not require permission from the institutional review board (IRB), as this was a retrospective observational study.

## Conclusions

GBC, though rare, is the most common malignancy of the pancreaticobiliary tract seen among females of North India. There is a strong association between gallstones and GBC, which is attributed to chronic irritation of the mucosa initiating the reparative-regenerative and dysplastic sequence. In our study, almost 40% of the total malignant cases were diagnosed incidentally. The detection of high-grade intraepithelial lesions and IGBC is difficult, despite advances in imaging modalities and a significant number of them are still missed despite meticulous gross examination. The selective approach of HPE of the gallbladder is cost-effective, especially in resource-constrained countries. However, since GBC carries a poor prognosis, timely management can prevent significant mortality, especially in the early stages of cancer. It is, therefore, imperative that HPE of all the surgically resected gallbladder specimens should be mandatory, especially in high-risk zones.
